# Melatonin in animal husbandry: functions and applications

**DOI:** 10.3389/fvets.2024.1444578

**Published:** 2024-09-02

**Authors:** Ruohan Zhao, Yicheng Bai, Fangxiao Yang

**Affiliations:** ^1^College of Animal Science and Technology, Yunnan Agricultural University, Kunming, Yunnan, China; ^2^College of Animal Science and Veterinary Medicine, Yunnan Vocational and Technical College of Agriculture, Kunming, Yunnan, China

**Keywords:** melatonin, reproductive performance, antioxidation, immune regulation, animal application

## Abstract

Melatonin (N-acetyl-5-methoxytryptamine) is an essential small molecule with diverse biological functions. It plays several key roles, including regulating the secretion of reproductive hormones and the reproductive cycle, enhancing the functionality of reproductive organs, improving the quality of sperm and eggs, and mitigating oxidative stress in the reproductive system. Melatonin effectively inhibits and scavenges excess free radicals while activating the antioxidant enzyme system and reduces the production of inflammatory factors and alleviates tissue damage caused by inflammation by regulating inflammatory pathways. Additionally, melatonin contributes to repairing the intestinal barrier and regulating the gut microbiota, thereby reducing bacterial and toxin permeation. The use of melatonin as an endogenous hormone in animal husbandry has garnered considerable attention because of its positive effects on animal production performance, reproductive outcomes, stress adaptation, disease treatment, and environmental sustainability. This review explores the characteristics and biological functions of melatonin, along with its current applications in animal production. Our findings may serve as a reference for the use of melatonin in animal farming and future developmental directions.

## 1 Introduction

Antibiotics play a crucial role in animal husbandry by promoting growth and preventing diseases, but their misuse has led to a series of problems. Antibiotics overuse fosters bacterial resistance, reducing treatment efficacy in humans and animals (1). Furthermore, antibiotic residues in animal products entering the food chain pose health risks, disrupting environmental microbial balance and affecting ecosystem stability (2). Excessive antibiotic use also adversely impacts animal health, disrupts the gut microbiota balance, reduces digestive absorption, and prompts adverse drug reactions (3, 4). Subsequently, many nations have implemented stringent regulations governing antibiotic use in poultry and livestock production, prompting increased research focus on alternative and enhanced additives.

Melatonin is an amine hormone primarily secreted by the pineal gland in the brain, but it is also produced in other tissues such as the gastrointestinal tract, retina, skin, thymus, and immune cells (5). The melatonin secreted by the pineal gland plays a crucial role in regulating circadian rhythms, while extra-pineal melatonin possesses anti-inflammatory, antioxidant, and mitochondrial protective properties (6–10). Melatonin receptors—MT1, MT2, and MT3—are widely distributed throughout the human body and perform various physiological functions (11, 12). MT1 regulates the biological clock, MT2 controls periodic melatonin activity, and MT3, part of the quinone reductase family, aids in detoxification. As members of the G protein-coupled receptor family on the cell surface, these receptors facilitate signal transduction, significantly influencing physiological and pathological cellular processes (13, 14).

Melatonin, through interactions with MT1 and MT2 receptors, modulates circadian rhythms, immune responses, antioxidation, neuroprotection, anti-aging, reproductive functions, and cell physiology (15–19). In humans, exogenous melatonin exhibits diverse effects, offering potential benefits in animal husbandry, including circadian regulation, health enhancement, stress reduction, and disease prevention (20). Despite the recognized roles of melatonin, current research has not fully explored its multifaceted effects across different animal species and production systems. The existing studies often focus on isolated functions of melatonin without considering its potential synergistic effects with other management practices. Furthermore, there is a lack of comprehensive reviews that integrate recent findings on melatonin’s impact on various aspects of animal production performance, reproductive outcomes, and stress adaptation. This review provides an overview of the physicochemical properties and biological functions of melatonin, highlighting the latest research findings on its potential to improve health and productivity in animal husbandry and laying the groundwork for prospective applications in animal production.

## 2 Structure and physicochemical properties of melatonin

Melatonin is an indoleamine compound with a molecular formula of C_13_H_16_N_2_O_2_, a relative molecular mass of 232.28, and a relative density of 1.175 g/cm^3^. It is sparingly soluble in water but readily soluble in propylene glycol and 2-hydroxypropyl-beta-cyclodextrin (21–23). Melatonin exists as a white crystalline powder with a melting point of approximately 116–118°C and a boiling point of 374.44°C. It has a relatively short half-life (30–50 min) and low oral bioavailability (9–33%) (24). Following intravenous or oral administration, melatonin is rapidly metabolized primarily by the liver and kidneys (25). Originally extracted from bovine brain pineal glands in 1917, the name “melatonin” stems from its ability to lighten frogs’ dark skin.

**Figure 1:**
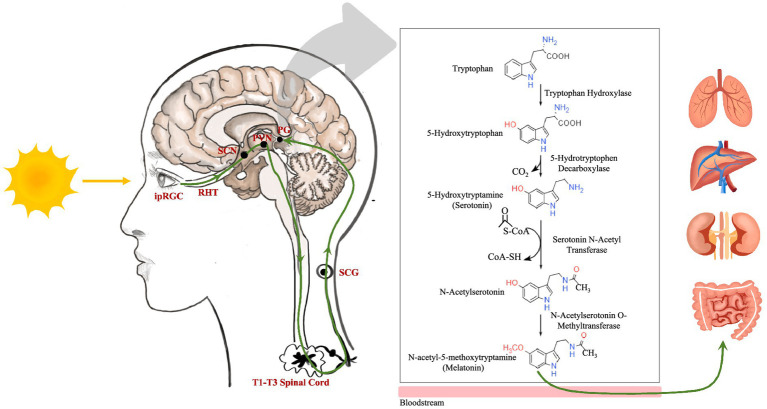
The neuroanatomical pathway of light stimulation on the pineal gland and the melatonin metabolic pathway. ipRGC, intrinsically photosensitive retinal ganglion cell; RHT, retinohypothalamic tract; SCN, suprachiasmatic nucleus; PVN, paraventricular nucleus; SCG, superior cervical ganglion; PG, pineal gland.

Melatonin is synthesized predominantly in the pineal gland in response to light exposure. Intrinsically photosensitive retinal ganglion cells receive light during the day and initiate a neural signal cascade via the retinohypothalamic tract. This neural pathway involves the suprachiasmatic nucleus, paraventricular nucleus, brainstem, spinal cord (T1–T3 levels), and superior cervical ganglion, culminating in the pineal gland. Synthesis begins when tryptophan is converted to 5-hydroxytryptophan by tryptophan hydroxylases. Subsequently, 5-hydroxytryptophan decarboxylase removes CO_2_ from 5-hydroxytryptophan to produce 5-hydroxytryptamine, also known as serotonin. Serotonin is transformed into N-acetylserotonin through serotonin N-acetyl transferase, aided by acetyl coenzyme A. Finally, N-acetylserotonin O-methyltransferase (ASMT) catalyzes the methylation of N-acetylserotonin to melatonin (Figure 1) (17, 18). Melatonin synthesis typically commences after sunset as the pineal gland converts serotonin to melatonin, releasing it into the bloodstream and impacting various organs (Figure 1). In addition, mitochondria from various organs can produce melatonin for localized use (26).

## 3 Biological function of melatonin

### 3.1 Antioxidation

During metabolism, animals generate reactive oxygen species (ROS), including hydrogen peroxide, hydroxyl radicals, and superoxide anions. Elevated ROS levels under stress or disease conditions can inflict damage on proteins, lipids, and DNA, disrupt cell membranes, and compromise immune function (27). Melatonin, functioning as a robust antioxidant, effectively counters these detrimental effects by neutralizing free radicals and enhancing the production of antioxidant enzymes, such as superoxide dismutase (SOD), glutathione peroxidase (GSH-Px), and catalase (CAT) (15, 28, 29). Numerous animal studies have demonstrated the ability of melatonin to mitigate oxidative damage through the activation of specific signaling pathways (Table 1). In a mouse model of testicular lipotoxicity, melatonin suppressed ROS production by activating the sirtuin 1 (SIRT1) signaling pathway. This action reduces the endoplasmic reticulum stress response (p-IRE1, p-PERK, and ATF4) and protein expression linked to apoptosis (B-cell lymphoma 2 [Bcl-2], Bcl-2-associated X [Bax], Caspase3, Caspase12, and CHOP), thereby alleviating oxidative damage in the testes (30). Similarly, melatonin exhibited protective effects against oxidative stress-induced damage in various animal models, including oocytes, renal tubular epithelial cells, brain cells, and myeloid cells (31–35). The protective mechanisms of melatonin involve the upregulation of key antioxidant response factors. For example, it activates the nuclear factor erythroid2-related factor 2 (Nrf2), reinforcing the cell’s antioxidant capability against oxidative stress (33, 36). Additionally, melatonin promotes reactions between glutathione (GSH), oxygen-free radicals, and other harmful oxidative substances, transforming them into harmless compounds (35). Moreover, it stabilizes mitochondrial membrane potential and enhances mitochondrial function, thereby mitigating mitochondrial dysfunction and apoptosis (37–39). In summary, melatonin counteracts oxidative stress-induced damage by scavenging reactive oxygen species, enhancing mitochondrial function, inhibiting lipid peroxidation, and preserving cell membrane fluidity, among other mechanisms.

**Table 1:** The antioxidant effects of melatonin.

Function	Target	Model	Dose of melatonin	Mode of action	References
Antioxidation	10 μM copper and 100 μM Que. or EGCG	Redox system	1 mM	Melatonin decreases hydroxyl radical formation and DNA damage by chelating copper to prevent the formation of hydroxyl radical.	(29)
Antioxidation	Male mice testicular tissues	Lipotoxicity	10 mg/kg	Modulating the SIRT1 signaling pathway decreases p-Nrf2, MDA, and ROS.	(30)
Antioxidation	Spermatogonia	Lipotoxicity	1 μmol/L	Modulating the MT/SIRT1/FoxO1 signaling pathway increases the activity of MnSOD and CAT, while decreasing Ac-FoxO1/FoxO1 and ROS.	(30)
Antioxidation	Mouse oocyte	Deoxynivalenol	1 × 10^−7^ M	Decreases GSH-Px, SOD, ROS.	(31)
Antioxidation	Oocytes from aged female mice	Aging	30 mg/kg	Increasing the activity of G6PDH, NADPH, and GSH leads to a decrease in ROS.	(32)
Antioxidation	Mouse tubular epithelial cells	Acute kidney injury	1 mM	Modulating the Nrf2/Slc7a11 signaling pathway increases the activity of GPX4, SOD, and GSH-Px, while decreasing MDA.	(33)
Antioxidation	Male mice	Repetitive mild traumatic brain injury	20 mg/kg	Regulation of p-AMPK/p-CREB signaling pathways. Decreases in ROS, iNOS, Cox-2.	(34)
Antioxidation	Nucleus pulposus cells	Oxidative stress	1 μM	Increase activity of SOD, GSH. Decreases in ROS, MDA.	(35)
Antioxidation	Hippocampal dentate gyrus region of the postnatal day 7 rat brain	Oxidative stress	10 mg/kg	Regulating the SIRT1/Nrf2 signaling pathways decreases Cox-2 and iNOS.	(36)

Que, quercetin; EGCG, (−)-epigallocatechin-3-gallate; SIRT1, silent information regulator sirtuin 1; p-Nrf2, phosphorylated nuclear factor erythroid 2-related factor 2; MDA, malondialdehyde; ROS, reactive oxygen species; MT, melatonin; FoxO1, forkhead box O1; MnSOD, manganese superoxide dismutase; CAT, Catalase; Ac-FoxO1, acetylated forkhead box O1; GSH-Px, glutathione peroxidase; SOD, superoxide dismutase; G6PDH, glucose-6-phosphate dehydrogenase; NADPH, nicotinamide adenine dinucleotide phosphate hydrogen; GSH, glutathione; Nrf2, nuclear factor erythroid 2-related factor 2; Slc7a11, solute carrier family 7a member 11; GPX4, glutathione peroxidase 4; p-AMPK, phosphorylated AMP-activated protein kinase; p-CREB, phosphorylated cAMP response element-binding protein; iNOS, inducible nitric oxide synthase; Cox-2, cyclooxygenase-2.

### 3.2 Immune regulation

Immune stimulation is a critical defense strategy against infections, inflammation, and tumors. The removal of the pineal gland leads to immune suppression by impairing melatonin synthesis, while oral melatonin supplementation promptly restores immune system functionality (40, 41). Melatonin replacement therapy enhances immune memory and antibody responses, maximizing immune system efficacy (42). This suggests the potential of melatonin in immune enhancement by reshaping thymus function and promoting T-cell generation, which is pivotal for combating microbial invasion and sustaining immune system integrity (43).

Melatonin exhibits anti-inflammatory effects through various mechanisms (44–46) (Table 2). It blocks the nuclear factor-κB (NF-κB)/gasdermin D signaling pathway, reducing the expression of inflammatory genes (*IL-1β*, *IL-6*, and *IFN-γ*), inhibiting inflammasome activation (NLR family pyrin domain-containing 3 [NLRP3] and ASC), and mitigating the release of inflammatory cytokines. These actions alleviate lipopolysaccharide (LPS)-induced inflammation in adipose tissue (47). In spinal cord injury models, melatonin suppresses NLRP3 inflammasome activation by activating the Nrf2 pathway and reducing pro-inflammatory factor secretion (48). In acute kidney injury models, melatonin reduces pro-inflammatory cytokine expression (IL-1β, TNF-α, MCP-1, and IL-6), enhances mitochondrial biogenesis protein expression (PGC1α and Tfam), and increases mitochondrial uncoupling proteins, alleviating kidney damage (33). Additionally, melatonin has significant potential as an adjunctive treatment for sepsis. It protects organ function by reducing inflammation, oxidative stress, endoplasmic reticulum stress, and apoptosis, while maintaining mitochondrial function and modulating various physiological pathways (49–51). Furthermore, melatonin produced by the lungs acts as a barrier against severe acute respiratory syndrome coronavirus 2, triggering immune responses, antibody production, and thwarting the virus from entering epithelial cells (52). In summary, melatonin offers promising benefits in bolstering the immune system, with its roles in immune enhancement, anti-inflammatory effects, and potential in combating specific diseases and infections.

**Table 2:** The immune regulation effect of melatonin.

Function	Target	Model	Dose of melatonin	Mode of action	Reference
Immune regulation	Male mice	Pinealectomy mice	40 mg/kg	Recovery of IL-2, IL-4, IL-6, IL-10, IL-17, and IFN-γ expression levels within 2 weeks.	(40)
Immune regulation	Rat	Ischemia/reperfusion	10 mg/kg	By inhibiting the TLR signaling pathway, the expression levels of MyD88, TNF-α, IL-6, and iNOS are reduced.	(44)
Immune regulation	Male rat	Colitis	10 mg/kg	Reduce the activity of IL-1, TNF-α, and NO.	(45)
Immune regulation	Human synovial fibroblasts	Rheumatoid arthritis	1 mM	Inhibition of TNF-α and IL-1β production by downregulating PI3K/AKT, ERK, NF-κB signaling pathways, and overexpression of miR-3150a-3p.	(46)
Immune regulation	Male mice white adipose tissue	Inflammation	20 mg/kg	Blockade of the NF-κB/GSDMD signaling pathway. Decrease in NLRP3, IL-1β, IL-6, IFN-γ, ASC, Caspase1, and Caspase3.	(47)
Immune regulation	Mice	Spinal cord injury	30 mg/kg	Inhibiting the activation of NLRP3 inflammasomes through the Nrf2/ARE signaling reduces the secretion of pro-inflammatory factors (ASC, caspase-1, and IL-1β).	(48)
Immune regulation	Mouse tubular epithelial cells	Acute kidney injury	1 mM	Modulation Nrf2/Slc7a11 signaling pathway. Inhibit the expression of pro-inflammatory cytokines IL-1β, TNF-α, MCP-1, and IL-6.	(33)
Immune regulation	Male mice	Sepsis-induced acute kidney injury	30 mg/kg	By activating SIRT3, the deacetylation of TFAM at the K154 site is mediated, thereby enhancing mitophagic flux.	(50)
Immune regulation	Mice	Sepsis-induced acute lung injury	30 mg/kg	Upregulate OPTN and ZO-1, downregulate STAT3 and TNF-α.	(51)

IL-2, interleukin-2; IL-4, interleukin-4; IL-6, interleukin-6; IL-10, interleukin-10; IL-17, interleukin-17; IFN-γ, interferon gamma; TLR, toll-like receptor; MyD88, myeloid differentiation primary response 88; TNF-α, tumor necrosis factor alpha; IL-1, interleukin-1; NO, nitric oxide; IL-1β, interleukin 1 beta; PI3K, phosphoinositide 3-kinase; AKT, protein kinase B; ERK, extracellular signal-regulated kinase; NF-κB, nuclear factor-kappaB; GSDMD, gasdermin D; NLRP3, NOD-like receptor family pyrin domain containing 3; ASC, apoptosis-associated speck-like protein containing a CARD; ARE, antioxidant response element; MCP-1, monocyte chemoattractant protein-1; SIRT3, sirtuin 3; TFAM, transcription factor A, mitochondrial; OPTN, optineurin; ZO-1, zonula occludens-1; STAT3, signal transducer and activator of transcription 3.

### 3.3 Nerve protection

Melatonin plays a pivotal role in neuroprotection by maintaining neural function, preventing damage, fostering regeneration, and preventing neurodegenerative diseases (53). Moreover, melatonin regulates cell proliferation and neuronal differentiation in neural stem/progenitor cells, enhancing the maturity of neural precursor cells and the development of new neurons (54, 55). These neuroregulatory effects are particularly beneficial under conditions such as stress, anxiety, depression, and ischemic brain injury (56–61) (Table 3). In mouse brain injury models, melatonin regulates energy utilization through the phosphorylated 5’AMP-activated kinase/phospho-cyclic adenosine monophosphate (cAMP) response element-binding signaling pathway. It diminishes the expression of pro-apoptotic factors (Bax, PARP-1, and Caspase3) and increases the expression of anti-apoptotic factors (Bcl-2), thereby shielding the brain from damage (34). Additionally, melatonin may attenuates age-related decline in brain function by improving lipid metabolism disturbances, restoring mitochondrial function, reducing neuronal damage, and moderating mechanisms associated with brain aging, regulated by core proteins (Mpst, Ccsap, Hdhd5, Rpl5, and Flna) (62). Furthermore, melatonin deficiency in endogenous secretions may disrupt mitochondrial homeostasis, releasing mitochondrial DNA (mtDNA) and triggering neuroinflammatory responses in the cytoplasm. Chronic inflammation, a hallmark of aging and neurodegenerative diseases, is mitigated by melatonin supplementation. In neurodegenerative disease mouse models, melatonin supplementation inhibits mtDNA release, preventing ROS damage, curtails neuroinflammatory pathway activation (cGAS/STING/IRF3), and reduces inflammatory cytokine production (IL-6, IL-18, IL-1β, IFN-α, and IFN-β). These actions mitigate the effects of brain aging and neurodegenerative pathologies (63). In summary, melatonin protects neurons, enhances neural function, and mitigates neurodegenerative alterations through diverse mechanisms implicated in neurodegenerative diseases.

**Table 3:** The neuroprotective effects of melatonin.

Function	Target	Model	Dose of melatonin	Mode of action	References
Nerve protection	Male mice	Depression	10 mg/kg	Restore coat state and grooming behavior, while reducing corticosterone levels.	(58)
Nerve protection	Male hamsters	Neuronal remodeling	20 μg	Increasing Period1 and Bmal1 leads to neuronal remodeling.	(59)
Nerve protection	Alzheimer’s disease transgenic mice	Alzheimer’s disease	0.5 mg	Restoration of mitochondrial respiratory rates, membrane potential, and ATP levels.	(60)
Nerve protection	Male rat	Alzheimer’s disease	3 mg/kg	Improved memory impairment, decreased phosphorylation levels of tau at Ser199/Ser202 and Ser396/Ser404, reduced expression of SMI31 and MDA, and increased levels of SOD and GSH-Px.	(61)
Nerve protection	Male mice	Repetitive mild traumatic brain injury	20 mg/kg	Regulation of p-AMPK/p-CREB signaling pathways. Improved motor function, decreased levels of BACE-1, APP, and Aβ.	(34)
Nerve protection	Male mice	Aging	3 mg/kg	By modulating core proteins (Mpst, Ccsap, Hdhd5, RPL5, and Flna), melatonin alleviates lipid dysregulation, restores mitochondrial function, and reduces neuronal damage.	(62)
Nerve protection	Transgenic mice	Neurodegenerative diseases	5 μM	mtDNA, cGAS, IL-6, STING, IL-1β, IRF3, IL-18, pNF-κB, IFN-α, IFN-β, and Caspase1 levels decreased.	(63)

Period1, period circadian regulator 1; Bmal1, brain and muscle Arnt-like protein-1; Ser, serine; SMI31, non-phosphorylated neurofilament heavy polypeptide; BACE-1, beta-secretase 1; APP, amyloid precursor protein; Aβ, amyloid beta; Mpst, mercaptopyruvate sulfurtransferase; Ccsap, centriole, cilia and spindle-associated protein; Hdhd5, haloacid dehalogenase-like hydrolase domain-containing protein 5; RPL5, ribosomal protein L5; Flna, filamin A; mtDNA, mitochondrial DNA; cGAS, cyclic GMP-AMP synthase; STING, stimulator of interferon genes; IRF3, interferon regulatory factor 3; IL-18, interleukin 18; IFN-α, interferon alpha; IFN-β, interferon beta.

### 3.4 Anti-aging

Aging is a progressive change within biological organisms, characterized by mitochondrial dysfunction, immune system damage, oxidative stress, and other related changes (64). The anti-aging effects of melatonin are primarily reflected in its protective role on mitochondrial function and its ability to alleviate mitochondrial dysfunction and cellular aging (38, 65, 66) (Table 4). Melatonin is found at higher concentrations in mitochondria within cells, where it effectively scavenges free radicals and reduces oxidative damage. According to the free radical theory of aging, high levels of melatonin help slow the onset and progression of aging and related diseases (38). Long-term administration of melatonin to aged rats can protect mitochondrial cytochrome c and 2′,3′-cyclic nucleotide 3′-phosphodiesterase levels by preventing the opening of the mitochondrial permeability transition pore, thereby enhancing mitochondrial function (67). Additionally, melatonin prevents mitochondrial dysfunction and cellular aging by limiting the oxidation of mitochondrial cardiolipin (68). Long-term intake of melatonin can also effectively prevent oxidative stress damage in the mitochondria of the heart and diaphragm in aging mice (69). Furthermore, exogenous melatonin administration has been shown to increase the expression of Bcl-2, glutathione peroxidase (GPx), and glutathione S-transferase (GST) in elderly rats, reduce the release of carbon monoxide (CO) and nitric oxide (NO), and lower the levels of cytochrome C, caspases, and lipid peroxidation (LPO), thereby contributing to melatonin’s anti-aging effects (65, 66).

**Table 4:** The anti-aging effects of melatonin.

Function	Target	Model	Dose of melatonin	Mode of action	References
Anti-aging	Male rat	Aging	7 mg/kg	Preventing mPTP opening, inhibiting the release of cytochrome c and CNPase from mitochondria, and increasing Ca^2+^ content.	(67)
Anti-aging	Cardiac and diaphragmatic of male mice	Aging	10 mg/kg	Decreased LPO, with increased GSH and GRd activity.	(69)
Anti-aging	Wistar rats and SAMP8 (senescence prone) mice	Aging	1 mg/kg and 10 mg/kg	Increase the expression of ATP, Bcl-2, GPx, and GST; decrease the release of NO and CO; reduce cytochrome c levels; and lower levels of subcutaneous fat, nucleic acid fragmentation, caspases, and LPO.	(65)
Anti-aging	Mice with LPS-induced DLB	Inflammation	500 mg/kg	Suppression of NLRP3 expression and IL-1β cleavage inhibits pyroptosis, the production of mitochondrial and cytosolic ROS, and NF-κB signaling.	(76)
Anti-aging	Male Wistar rats	Inflammation	5 mg/kg	Reduce the levels of IL-4, IL-10, TNF-α, O_2_^−^, and H_2_O_2_ production; increase catalase and SOD; and inhibit NF-κB activation.	(77)
Anti-aging	Rats	Acute kidney injury	10 mg/kg	Regulate oxidative stress through the AKT/FOXO and Bax/Caspase-3 signaling pathways, inhibit apoptosis, lower serum levels of blood urea nitrogen and creatinine, upregulate Klotho protein, and reduce the phosphorylation ratios of AKT and FOXO.	(78)
Anti-aging	Bone marrow-derived macrophages	Acute lung injury	30 mg/kg	Promote the transition from M1 to M2 type, inhibit pyroptosis, suppress the NLRP3/GSDMD pathway, and reduce levels of MDA, LDH, and ROS.	(79)
Anti-aging	Male albino rats	Aging	10 mg/kg	Activate the DJ-1/Nrf2 signaling pathway, inhibit the p53/Bax apoptotic pathway, lower levels of p53, Bax, TNF-α, IFN-γ, IL-6, IL-8, and serum IgA, increase serum IgE, and elevate mRNA expression levels of DJ-1 and Nrf2.	(81)
Anti-aging	Swiss albino male mice	Aging	2 mg/L	Antigenotoxicity, antimutagenic activity, reduction of DNA damage, and increased levels of APE1 and OGG1 repair enzymes.	(82)

mPTP, mitochondrial permeability transition pore; CNPase, 2′,3′-cyclic nucleotide 3′-phosphodiesterase; LPO, lipid peroxidation; GRd, glutathione reductase; Bcl-2, B-cell lymphoma 2; GPx, glutathione peroxidase; GST, glutathione S-transferase; CO, carbon monoxide; O_2_^−^, superoxide anion; H_2_O_2,_ hydrogen peroxide; FOXO, forkhead box O; Bax, Bcl-2-associated X; LDH, lactate dehydrogenase; DJ-1, protein deglycase 1; IL-8, interleukin-8; IgA, immunoglobulin A; IgE, immunoglobulin E; APE1, apurinic/apyrimidinic endonuclease 1; OGG1, 8-oxoguanine DNA glycosylase 1.

Extensive data indicate a close relationship between oxidation and inflammation, as excessive oxidative stress can trigger inflammatory responses, with ROS being considered as effectors of inflammation (70, 71). Clinical data confirm that chronic inflammation promotes aging, with increased levels of chronic inflammation being referred to as inflammaging (72–75). Melatonin may also upregulate Nrf2 and downregulate NF-κB, thereby alleviating cellular-level inflammatory processes (76, 77). Similarly, melatonin upregulates the expression of the anti-aging protein Klotho, stimulates the release of anti-inflammatory cytokines (IL-4 and IL-10), and promotes the polarization of macrophages from a pro-inflammatory (M1) phenotype to an anti-inflammatory (M2) phenotype (78, 79). Additionally, melatonin inhibits various pro-inflammatory events, such as amyloid toxicity, NO release, cyclooxygenase-2 (Cox-2) and NLRP3 inflammasome activation, toll-like receptor 4 (TLR4) and mammalian target of rapamycin (mTOR) signaling, as well as the release of senescence-associated secretory phenotype cytokines. It also activates the protein deglycase 1 (DJ-1)/Nrf2 antioxidant signaling pathway and inhibits the p53/Bax apoptotic pathway (80, 81). Importantly, exogenous melatonin can increase levels of apurinic/apyrimidinic endonuclease 1 (APE1) and 8-oxoguanine DNA glycosylase 1 (OGG1) in mice, facilitating DNA damage repair (82). Thus, melatonin demonstrates pharmacological regulation of aging and has been proposed as a molecule with potential anti-aging effects, with the possibility of extending lifespan through promoting healthy aging.

### 3.5 Other biological functions

In addition to its multifaceted roles, melatonin has various beneficial effects, including the regulation of blood sugar, reduction of blood lipids, restoration of intestinal barrier function, and modulation of gut microbiota composition. Importantly, melatonin exerts an extensive effect on glucose homeostasis (83). In diabetic rodent models, melatonin and insulin co-administration enhance the sensitivity of white adipose tissue to insulin and improve blood glucose control (84). Melatonin plays a dual role by regulating glucose metabolism and safeguarding pancreatic β-cells. In addition, studies have indicated its potential in mitigating apoptosis in pancreatic β-cells exposed to high glucose conditions, reducing the expression of aging-related proteins (β-Galactosidase). Moreover, melatonin augments endogenous antioxidant defenses (CAT and Mn-SOD), enhances insulin secretion in response to glucose stimulation, and mitigates cell apoptosis and stress-induced premature aging in pancreatic β-cells caused by glucotoxicity and lipotoxicity (85). These orchestrated actions extend β-cell lifespan and fortify their function, underscoring melatonin’s significance in glucose regulation.

In diverse animal models, melatonin demonstrates an inhibitory effect on weight gain and phenotypes linked to visceral fat accumulation, particularly in models with high-fat and high-sugar diets (86). Melatonin significantly promotes fat cell lipolysis by increasing lipolysis-related gene and protein expressions, including hormone-sensitive lipase (HSL), adipocyte triglyceride lipase (ATGL), and perilipin 1, predominantly through the MT2 receptor (87).

Intestinal health is crucial for the overall well-being of animals. Melatonin exhibits a significant impact on intestinal damage by elevating the expression of intestinal tight junction proteins (ZO-1, Occludin, and Claudin-1), reducing intestinal permeability, and modulating gut microbiota composition by decreasing the abundance of *Firmicutes* and increasing the abundance of *Bacteroidetes* phyla (88). Overall, the broad application of melatonin across various domains signifies its pivotal role in regulating glucose and lipid metabolism, intestinal health, and the composition of the gut microbiota.

After exploring melatonin’s various biological functions such as antioxidation, immune modulation, and neuroprotection, it is evident that these functions could may enhance various aspects of animal husbandry. Melatonin’s abilities to mitigate oxidative stress, boost immune responses, protect neural tissues, and regulate metabolic processes provide a solid foundation for its application in animal production. Next, we will delve into how these biological functions can be translated into practical applications across different animal species, aimed at improving productivity, reproductive capabilities, and overall health and welfare of livestock.

## 4 Potentials of melatonin in animal production

### 4.1 Effect of melatonin on animal production performance

#### 4.1.1 Poultry

Although the exact mechanisms underlying melatonin’s effects on poultry remain unclear, research suggests its potential impact on growth through various pathways, including the regulation of growth hormone (GH) secretion and metabolism. Melatonin influences the release and action of GH by promoting growth hormone-releasing hormone (GHRH) secretion and inhibiting somatostatin secretion (89, 90). Moreover, melatonin may modulate poultry growth by regulating the expression of growth-related genes and cell proliferation and differentiation. Studies have revealed that melatonin, which binds to the melatonin receptor subtypes Mel1b and Mel1c in the pituitary gland, triggers pituitary-specific transcription factor-1 expression in the anterior pituitary cells, thus boosting growth hormone secretion and fostering growth (91). Importantly, melatonin-mediated green light induction can engage diverse signaling pathways. The Mel1b receptor acts via the adenylate cyclase/protein kinase A (PKA)/extracellular signal-regulated kinase 1/2 (ERK1/2) pathway, and the Mel1c receptor, regulated by ERK1/2, induces GH secretion in the chicken pituitary gland (92). Additionally, *in vitro* experiments demonstrated that exogenous melatonin augments chicken liver cell proliferation and insulin-like growth factor 1 (IGF-1) secretion (93). However, the effects of melatonin on poultry body weight vary across studies and have not been consistently reported. Some studies have revealed that melatonin can reduce poultry feed intake, enhance feed efficiency, and elevate body weight under certain conditions, though results vary based on environmental factors.

For instance, oral administration of melatonin to broilers under continuous 24-h lighting and hot, dry conditions resulted in increased live weight and average weight gain but reduced feed consumption (94). Similar findings have demonstrated reduced feed consumption in broilers treated with melatonin under different lighting conditions (95). Additionally, melatonin supplementation in heat-stressed quails increased the final body weight and liver weight, highlighting the potential benefits of improving poultry growth performance under heat-stress conditions (96). However, in broilers raised under suitable temperature and lighting conditions, melatonin supplementation had a minimal effect on body weight and feed consumption, except for a reduction in the incidence of sudden death syndrome (97). Further investigations are warranted to elucidate the specific mechanisms underlying the effects of melatonin on poultry welfare and productivity. Nevertheless, its potential positive effects warrant continued attention and exploration.

#### 4.1.2 Swine

Research on the impact of melatonin on pig production performance is limited, but evidence suggests that melatonin may have negligible effects in this context. However, melatonin is lipophilic and can traverse various subcellular compartments, promoting muscle development through multiple mechanisms (98). Exogenous melatonin supplementation stimulates fat breakdown and metabolism while promoting the differentiation of white adipocytes into brown adipocytes, thereby reducing fat content (99–101). *In vitro* experiments show that melatonin significantly promotes adipogenic differentiation of preadipocytes by increasing the expression of CCAAT/enhancer binding protein α (C/EBPα) and peroxisome proliferator-activated receptor γ (PPARγ) (102). Other studies reported that melatonin reduces fat content by promoting lipid metabolism in porcine oocytes (103). Importantly, melatonin promotes intramuscular fat breakdown by activating the PKA/ERK1/2 signaling pathway, enhancing mitochondrial biogenesis and mitochondrial respiration, and inhibiting preadipocyte proliferation in pig muscles (104). In weaned piglets, melatonin supplementation did not significantly affect growth performance but increased longissimus dorsi muscle weight and eye muscle area. Additionally, melatonin enhanced the expression of genes related to cell differentiation and muscle fiber development (*PAX7*, *MYOG*, *MYHC IIA*, *MYHC IIB*, *IGF-1*, and *IGFBP5*), modulated lipid metabolism (upregulating *COX6A*, *COX5B*, and *CPT2*, and downregulating *PPARG*, *ACC*, and *FABP4*), and activated mitochondrial function in muscle, thereby reducing fat deposition in muscle (105).

Melatonin also positively affects the intestinal health of pigs by regulating intestinal motility, expression of barrier integrity-related genes, and influencing absorption function and gut microbiota. Melatonin increased *Actinobacteria* abundance and decreased *Selenomonadales* abundance, enhancing piglet growth (106). Moreover, in *in vitro* embryo culture, exogenous melatonin enhances embryo development quality, reduces oxidative stress, enhances DNA integrity, and improves *in vitro* embryo development efficiency, especially during the maturation and fertilization stages (107). Although melatonin positively influences muscle development and fat metabolism in pigs, its impact on growth performance remains limited and requires further study for lean pig cultivation.

#### 4.1.3 Ruminants

Supplementing pregnant ewes with melatonin significantly enhances twin lamb survival rates, particularly during prolonged labor, enhancing their hypoxia tolerance (108). Melatonin supplementation also positively affects the health and growth performance of ewes and lambs (109, 110). It improves fetal oxygen supply, increases birth weight, enhances twin lamb vitality, elevates immunoglobulin G concentrations in the colostrum, enhances colostrum quality, and increases milk production, subsequently benefiting lamb growth (111, 112).

Similarly, postpartum ewes treated with melatonin exhibited increased weaning weight, average daily gain, and higher milk fat content, whereas milk protein and lactose remained unaffected (113). However, direct melatonin implantation in lambs does not significantly affect the growth rate (114). Nonetheless, melatonin implantation in lambs enhances muscle fiber and adipocyte cross-sectional areas, along with related indicators such as red blood cell count, testosterone, growth hormone, and immunoglobulin A. Transcriptome and microbiome analyses suggest that melatonin promotes lamb growth and development by modulating cell apoptosis signaling pathways and the gut microbiota (115).

Conversely, melatonin supplementation in pregnant and postpartum cows does not significantly affect calf weight, morphometric measurements, growth, metabolic factors, or subsequent bull reproductive characteristics. Although melatonin may influence milk yield and fat percentage, these differences are not significant (116). Nonetheless, research indicates the potential of melatonin to improve dairy herd parameters, increase milk nutritional value, elevate lactose and protein contents, and reduce somatic cell counts in milk (117). In summary, melatonin generally does not affect the growth performance of adult ruminants but may positively influence newborn calves and lambs.

### 4.2 Effect of melatonin on reproductive performance of animals

#### 4.2.1 Poultry

Melatonin influences poultry through various pathways, enhancing reproductive function and breeding performance. In male poultry, melatonin promotes the proliferation of chicken testicular supporting cells by activating the ERK/inhibin alpha subunit signaling pathway and increasing the expression of cell proliferation-associated genes and proteins (PCNA and CCND1) (118). Furthermore, melatonin mitigates oxidative stress-induced apoptosis in rooster testicular interstitial cells by activating the protein kinase B (AKT)/Nrf2 signaling pathway, thereby reducing apoptosis (119). It also alleviates glyphosate (GLY)-induced damage to chicken testicular interstitial cells and seminiferous tubule structure, as well as declines in sperm quality, by mitigating mitochondrial dynamics imbalance and inhibiting mitochondrial autophagy. This improves GLY-suppressed testicular hormone synthesis (120). Additionally, melatonin supplementation to frozen–thawed rooster semen significantly enhances post-thaw sperm motility, plasma membrane integrity, and mitochondrial activity. It also maintains sperm integrity and function by reducing LPO and DNA fragmentation (121, 122).

In female poultry, *in vitro* experiments indicated that melatonin, through receptor activation, regulates the mTOR signaling pathway, upregulating cell cycle-related proteins (cyclin D1) and anti-apoptotic proteins (Bcl-2) and downregulating the pro-apoptotic protein Caspase3 and autophagy-related proteins (Beclin1 and LC3-II), which promotes the proliferation of chicken granulosa cells (123). *In vivo* experiments with laying hens demonstrated that exogenous melatonin increases melatonin receptor expression in the ovaries by activating the mTOR signaling pathway. This upregulates the expression of downstream components of mTOR, S6 kinase, and 4E-binding protein 1, leading to enhanced follicle growth, extended physiological peak of egg production, and increased egg-laying rate (124). Specifically, supplementation with 10 mg melatonin in chickens at 360 and 550 days of age resulted in an 8.38 and 7.93% increase in egg-laying rates, respectively. This improvement in egg production is associated with elevated serum oestradiol-17β and reduced gonadotropin-inhibitory hormone receptors in the ovaries (125). These findings underscore the critical role of melatonin in poultry reproductive performance, providing essential scientific evidence for enhancing egg production in poultry.

#### 4.2.2 Swine

Melatonin plays crucial roles in oocyte maturation and embryonic development by enhancing these processes through multiple mechanisms. It upregulates the expression of genes related to lipid synthesis (*ACACA*, *FASN*, *PPARγ*, and *SREBF1*) and promotes the expression of lipolytic genes (*ATGL*, *CGI-58*, *HSL*, and *PLIN2*), thereby increasing fatty acid oxidation and improving mitochondrial biogenesis (peroxisome proliferator-activated receptor gamma co-activator 1 alpha [*PGC-1α*], *TFAM*, and *PRDX2*) to provide the energy required for oocyte development (103).

Additionally, melatonin improves the developmental rate and number of blastocyst cells in pig embryos, thereby promoting embryonic development (126, 127). Supplementing sows with melatonin in late pregnancy can yield multiple benefits, including increased litter size, enhanced birth survival rate, and higher weight, weaning weight, and survival rate of piglets. These effects may be achieved through the Nrf2 signaling pathway and upregulation of antioxidant genes [*MGST1*, *GSTM3*, and *GSTA4* (128)].

Melatonin also improves oocyte maturation and embryonic development by activating the sonic hedgehog (Shh) signaling pathway and upregulating the expression of related genes (*SHH*, *PTHC1*, *SMO*, and *GLI1*) (129). It promotes oocyte maturation by reducing granulosa cell apoptosis and stimulating estrogen synthesis (130, 131). In early pregnancy, melatonin improves the interaction between the uterus and conceptus by regulating SIRT1 and promoting the proliferation and migration of porcine trophoblast cells (132).

Moreover, melatonin regulates reproductive performance by modulating the release and synthesis of gonadotropin-releasing hormone and luteinizing hormone (LH) (133). It can counteract fungal toxins and toxic compounds, preventing oocyte maturation failure caused by ROS, improving the development of cloned embryos, and enhancing cloning efficiency (134–136). However, the effectiveness of melatonin in addressing seasonal breeding issues remains limited (137). In conclusion, melatonin promotes oocyte maturation and embryonic development, increases litter size and survival rates, and has antioxidant and protective characteristics, thereby playing a vital role in pig reproduction.

#### 4.2.3 Ruminants

Studies have demonstrated the significant enhancement of developmental ability in *in vitro* matured (IVM) cattle oocytes and embryos with melatonin (138). Specifically, melatonin aids in the recovery of meiosis during IVM of cattle oocytes without stimulating nuclear maturation processes, resulting in a higher proportion of blastocyst formation, both in the oocytes of adult cows and prepubertal donors (139).

Furthermore, melatonin improves embryo quality by augmenting the number of inner cell mass (ICM) cells and the ratio of ICM cells to total cells, indicating its positive influence on embryo differentiation and quality (140). Research has also revealed the expression of ASMT and the melatonin receptor MTNR1A in cattle oocytes and cumulus cells, with MTNR1B expressed exclusively in oocytes. Additionally, 10 and 50 ng/mL of melatonin significantly enhanced nuclear maturation and cumulus cell expansion and induced alterations in mitochondrial distribution patterns and ROS levels (141).

Importantly, melatonin also enhances the quality of thawed bull semen by increasing the average motion parameters, subpopulation structure, survival rate, and acrosomal integrity (142). In artificial insemination, melatonin improves the pregnancy rate and progesterone levels in female cattle, enhances uterine blood flow, and promotes placental development (143–145). For low-reproductive-season water buffaloes, melatonin injections significantly increase the ovulation rate, ovulation follicle diameter, and pregnancy rate (146).

Research involving melatonin implants in bulls revealed that the melatonin group exhibited higher hormone levels (FSH, LH, and testosterone) and melatonin concentrations than the control group. Simultaneously, bulls in the melatonin group showed improved sexual behavior scores, scrotal circumference, and testicular parameters (147). In summary, melatonin plays a crucial role in enhancing the reproductive protection and performance of ruminants, positively impacting the development and quality of oocytes and sperm, thereby facilitating the successful progression of the reproductive process.

### 4.3 Effects of melatonin on oxidative stress in animals

#### 4.3.1 Poultry

Poultry faces various oxidative stressors, such as ROS, peroxides, heavy metal ions, environmental pollutants, and heat stress, during rearing, which can adversely impact their health and productivity (148, 149). Melatonin serves as a protective antioxidant by neutralizing free radicals and reducing oxidative damage. A study demonstrated that injecting melatonin (500 mg/kg) into chicks increased melatonin content in tissues, including the intestine, kidney, liver, and red blood cells, by 75–1,300% compared to the control group. Furthermore, melatonin increased GSH-Px activity in these tissues by 22–134% (150). In laying hens, melatonin mitigates ovarian oxidative stress by modulating the SIRT1-P53/forkhead box O1 (FoxO1) pathway (21).

Additionally, the study found that melatonin can alleviate oxidative damage in chicken Leydig cells by activating the AKT–Nrf2 signaling pathway, resulting in increased antioxidant enzyme activity (119). Furthermore, melatonin significantly mitigates mycotoxin-induced oxidative stress in broiler chickens, reducing tissue damage markers (AST, ALT, and LPO) while increasing concentrations of antioxidant enzymes (SOD and CAT) in the serum. A 30% increase in the bursa weight of the Fabricius was also observed (151). Therefore, melatonin, as an antioxidant, holds significant potential in poultry farming, offering effective protective measures to improve poultry health. Nevertheless, further research is imperative to elucidate the mechanisms of the antioxidant action of melatonin in poultry, optimize application methods and dosages, and fully harness its potential benefits.

#### 4.3.2 Swine

The reproductive performance of boars closely correlates with their reproductive development and semen quality, which is frequently jeopardized by oxidative stress. Melatonin mitigates oxidative stress and cell apoptosis induced by tetrabromobisphenol A, reducing the disruption of the phosphatase and tensin homolog of chromosome 10/phosphatidylinositol 3-kinase (PI3K)/AKT signaling pathway and suppressing excessive ROS generation. Under oxidative stress conditions, it enhances antioxidant capacity by activating kelch-like ECH-associated protein 1/Nrf2 signaling, cell cycle, and lysosomal pathways. Additionally, melatonin increases the expression of heat shock protein 90 and stabilizes hypoxia-inducible factor-1α, effectively alleviating oxidative stress and cell apoptosis in support cells of boars induced by heat stress and chloroquine (152–154).

Melatonin also protects porcine oocytes from oxidative stress by maintaining cell morphology, reducing apoptosis, and delaying mitochondrial dysfunction (155). Melatonin also alleviates oxidative stress in porcine oocytes exposed to ochratoxin A, aflatoxin B1, heat stress, and other damaging factors by increasing antioxidant (GSH) levels, reducing apoptosis (SIRT1 and AKT2), and promoting cellular autophagy (by upregulating Bcl-2 and downregulating Bax, Atg7, Lc3, LC3B, and Caspase3) (156–159).

Pig embryo development is susceptible to various oxidative stress factors in the environment, including maternal factors, diet, drugs, diseases, and infections. Melatonin alleviates oxidative stress during *in vitro* embryonic development by activating the Nrf2/ARE signaling pathway and upregulating the expression of apoptosis-related genes (*MT2*, *Nrf2*, *UCHL*, *HO-1*, *SOD1*, and *Bcl-2*) (127). Additionally, melatonin reduces ROS production by promoting mitochondrial biogenesis (upregulating SIRT1 and PGC-1α), thereby rescuing early pig embryo development damage caused by pesticides such as rotenone (160).

In studies involving ischemia–reperfusion injury and allograft transplantation of porcine organs, melatonin significantly delayed the onset of rejection reactions, prolonged graft survival, and reduced oxidative stress (malondialdehyde [MDA] and 4-HDA) and inflammatory marker levels (pMAP and ITIH4) (161). Therefore, melatonin holds potential therapeutic value in transplantation. In conclusion, melatonin plays a crucial role in pig reproductive function, semen quality, embryo development, and the transplantation rejection response by inhibiting oxidative stress and promoting antioxidant capabilities.

#### 4.3.3 Ruminants

Melatonin effectively mitigates mycotoxin-induced oxidative stress and apoptosis in granulosa cells by inhibiting the p38 mitogen-activated protein kinase (MAPK) signaling pathway. This is accompanied by a reduction in the Bax/Bcl-2 ratio and increased levels of antioxidant enzymes (SOD and GSH-Px) (162). Similarly, melatonin protects bovine oocyte maturation and preimplantation embryo development from exposure to the herbicide paraquat by inhibiting the activation of the p38 MAPK signaling pathway. Melatonin restores abnormal levels of chromatin protein trimethylation of histone H3 lysine 4 and histone H3 lysine 9, regulates the expression of redox-related genes (such as decreasing thioredoxin-interacting protein [Txnip] and increasing Trx expression), and inhibits the expression of proapoptotic proteins (Caspase3 and Bax) (163, 164).

Furthermore, melatonin acts by activating the SIRT1/FoxO1 signaling pathway, reducing ROS levels and Ca^2+^ concentration, releasing cytochrome C, and increasing mitochondrial membrane potential (ΔΨm). It also promotes mitochondrial autophagy, alleviating oxidative stress-induced apoptosis and mitochondrial damage in bovine ovarian granulosa cells (165). Melatonin also protects cattle embryos from oxidative stress caused by hydrogen peroxide (H_2_O_2_) during *in vitro* culture, enhances the developmental quality of fertilized eggs, lowers intracellular ROS levels, and prevents mitochondrial dysfunction in zygotes (166, 167). Finally, melatonin alleviates heat stress in cattle exposed to high temperatures (168, 169). These findings underscore the critical role of melatonin in protecting ruminants from oxidative damage.

### 4.4 Effects of melatonin on animal immunity

#### 4.4.1 Poultry

In poultry farming, various environmental stressors, such as toxins, drugs, viruses, and diseases, are encountered daily. These factors can compromise immunity and impede the development of immune organs, ultimately resulting in immune dysfunction in poultry (170). Melatonin exerts several immunomodulatory effects in poultry, enhancing immune system function and resilience. A study demonstrated that supplementing the diet of young chickens with melatonin effectively alleviated duodenal inflammation induced by LPS. This effect is primarily achieved by inhibiting the TLR4 signaling pathway, reducing epithelial cell apoptosis, decreasing the expression of inflammatory cytokines (TNF-ɑ, IL-6, IL-4, and Caspase3), and improving the intestinal immune barrier function (mucin 2 [MUC2] and immunoglobulin A) (171). During embryonic development in broiler chickens, melatonin supplementation enhances the intestinal immune barrier by increasing goblet cell numbers and upregulating *MUC2* expression (172).

Moreover, *in vitro* experiments have demonstrated that melatonin promotes T lymphocytes in poultry peripheral blood, with minimal impact on B lymphocytes (173, 174). Green light with longer wavelengths stimulates photoreceptors in poultry retinas, influencing melatonin synthesis and release from the pineal gland. Compared to other light sources, green light stimulation increases the proliferation of B lymphocytes in the bursa of Fabricius by 16.49–30.83%. Green light also significantly upregulates the expression of the melatonin receptor subtypes Mel1a, Mel1b, and Mel1c (175, 176). Additionally, green light enhances the proliferation of T lymphocytes in the spleen by 2.46–6.83% by triggering the cAMP/PKA and phospholipase C/protein kinase C signaling pathways (177).

However, melatonin may produce varying effects on different types of immune cells and immune responses (174). Some studies have indicated that melatonin alone does not significantly affect lymphocyte proliferation in the chicken thymus, spleen, or bursa of Fabricius (178). Conversely, melatonin directly inhibits activated chicken lymphocytes *in vitro* through phytohemagglutinin stimulation, which may contribute to cell stimulation (179). In summary, the immunomodulatory effects of melatonin on poultry are multifaceted. It enhances poultry immune system function by inhibiting inflammatory responses, improving intestinal immune barrier function, and promoting lymphocyte proliferation. However, further research is required to better understand the specific mechanisms underlying the role of melatonin in immune modulation in poultry.

#### 4.4.2 Swine

Melatonin holds promise for immune regulation in pigs, particularly in immune challenges such as infections and inflammation. The immature gastrointestinal immune system of weaned piglets is susceptible to pathogenic infections such as enterotoxigenic *E. coli* (ETEC). ETEC infection affects the serotonin pathway in piglet macrophages, leading to reduced melatonin production. However, melatonin treatment can modify macrophage function, enhancing antimicrobial and bactericidal activities while reducing cell death. Additionally, melatonin pretreatment improves the ability of porcine macrophages to clear ETEC bacteria (180).

In a pig model of acute pancreatitis, melatonin therapy showed potential benefits, including reduced pancreatic acinar cell necrosis, adipose tissue necrosis, and edema, thereby improving pig adaptability and health scores (181). Furthermore, during early pregnancy, melatonin promotes the proliferation and migration of porcine trophectoderm and endometrial luminal epithelial cells via the SIRT1/PI3K/MAPK signaling pathway. Further, it also prevents pregnancy-related complications by reducing the production of pro-inflammatory factors and endoplasmic reticulum stress-sensitive proteins induced by LPS and tunicamycin (132). In summary, melatonin plays a crucial role in the immune regulation and health maintenance of pigs through various mechanisms under different physiological and pathological conditions. These findings offer valuable insights into its potential applications in pig farming.

#### 4.4.3 Ruminants

Melatonin exhibits promising potential in mitigating bovine mastitis, a prevalent inflammatory disease caused by bacterial infections that significantly impact dairy production and quality (182). Its multifaceted effects include the reduction of oxidative stress, inhibition of pro-inflammatory factors, and modulation of NF-κB signal transducers and activators of transcription (STATs), positioning it as a valuable candidate for alleviating bovine mastitis (183). In bovine mammary epithelial cells challenged with LPS-induced inflammation, melatonin mitigates the inflammatory response by inhibiting the cluster of differentiation 14/TLR4 signaling pathway. Specifically, it decreases the expression of pro-inflammatory cytokines (TNF-α, IL-1β, IL-6, and GM-CSF), chemokines (CCL2 and CCL5), and positive acute-phase proteins, while augmenting the expression of anti-inflammatory cytokines (IL-1Ra) and negative APP fibrinogen (184). Furthermore, melatonin inhibits *Staphylococcus aureus*-induced mastitis through the microRNA-16b/Yes-associated protein 1 pathway (185).

Melatonin enhances endometrial receptivity by reducing IL-6 levels and mitigating ammonia-induced inflammation and cell apoptosis by inhibiting the TLR4/NF-κB signaling pathway (186). In endometritis, melatonin effectively reduces the production of pro-inflammatory factors (IL-1β, IL-6, and TNF-α) in endometrial epithelial cells by inhibiting the activation of the NLRP3 inflammasome (187). Additionally, by activating membrane receptors MT1 and MT2, melatonin exerts anti-inflammatory effects on ovine epididymal epithelial cells by reducing the expression levels of pro-inflammatory cytokines and Cox-2 (188). Similar studies have found that melatonin, by activating membrane receptors MT1 and MT2 and activating the PI3K/AKT pathway, inhibits the LPS-induced inflammatory response in ovine endometrial epithelial cells (189). Furthermore, melatonin enhances sheep antibody titers against *A1* and *C* strains of *Dichelobacter nodosus* and the efficacy of the bovine viral diarrhea virus vaccine (190, 191). In summary, melatonin exerts anti-inflammatory and antiviral potential in ruminants and may offer a novel strategy for preventing bovine mastitis, potentially serving as an alternative to antibiotics in the treatment of this disease. Further research is warranted to explore the full therapeutic potential of melatonin in ruminant health management.

### 4.5 Application of melatonin in aquatic animal production

The inclusion of melatonin in the diet of *Cherax destructor* significantly improved weight gain, specific growth rate, and digestive enzyme activity. It also enhanced the activity of antioxidant enzymes, reduced oxidative stress, and improved immune parameters. The optimal dosage for dietary melatonin supplementation falls within the range of 75–81 mg/kg (192). In Asian sea bass, melatonin treatment increased body weight and survival rates, lowered blood glucose levels, and enhanced testosterone levels, promoting overall growth and health (193). However, dietary melatonin supplementation significantly reduced the growth performance of juvenile golden pompanos (194).

Melatonin facilitated fish oocyte maturation, increased antioxidant enzyme activity, reduced oxidative stress markers, and inhibited apoptosis (195, 196). In the ovaries of common carp, melatonin accelerated oocyte growth, alleviated oxidative stress, and exhibited stage-dependent effects on the reproductive cycle. It stimulated gonadal activity during the reproductive preparation stage and exerted anti-gonadal effects before and during spawning (197). Moreover, the addition of melatonin to the cryoprotective medium improved the post-thaw sperm quality of fish (198).

In aquatic animals, melatonin plays a crucial role in regulating Na^+^/K^+^-ATPase function, particularly during recovery and stress responses under hypoxic conditions (199). Melatonin improves the oxidative status of fish liver cells by activating the ERK/Akt and NF-κB/Nrf2 signaling pathways, thereby regulating heat shock factor expression. This protection prevents damage, reduces oxidative markers such as MDA levels, and inhibits the expression of pro-inflammatory cytokines (IL-1β, IL-6, IL-10, and TNF-α), ultimately mitigating stress-induced damage and inflammation (200, 201).

Melatonin exerts multiple protective effects on fish health under various environmental conditions. It effectively mitigates the damage and toxic effects of harmful substances such as 2,2,4,4-tetra-brominated diphenyl ether, lead, imidacloprid (a pesticide), polystyrene microplastics, and radiation by modulating different pathways, including the miR-140-5p/TLR4/NF-κB pathway, AMPK/SIRT1/PGC-1α axis, miR-17-5p/TXNIP axis, and peptidoglycan/P38 MAPK pathway. These findings underscore the significant protective role of melatonin in the health of aquatic organisms by reducing inflammation, oxidative stress, mitochondrial dysfunction, and apoptosis, providing robust support for biological well-being under environmental toxin exposure (202–209). In summary, melatonin significantly enhances the growth, health, and reproductive success of various aquatic species. Its protective effects against oxidative stress, inflammation, and environmental toxins highlight its potential as a valuable supplement in aquaculture.

### 4.6 Potential effect of melatonin on meat quality

Several studies have demonstrated melatonin’s significant efficacy in mitigating muscle atrophy (210). In the eyelid skin of elderly individuals, melatonin downregulates the age-promoting mechanistic target of rapamycin complex 1 (mTORC1) pathway and matrix metalloproteinase-1 (MMP-1) expression, while promoting the expression of transmembrane collagen 17A1 (COL17A1) and mitochondrial markers (such as TFAM, MTCO-1, and VDAC/porin). This modulation leads to increased elastin content and enhanced structural organization of elastin (211). Additionally, melatonin maintains the normal structure, weight, muscle fiber count, and function of aging muscles. It also enhances lactate production, prevents mitochondrial damage and the formation of tubular aggregates, reduces the percentage of apoptotic cells in aging muscles, and restores the frailty index (212, 213).

In animal production, meat is a significant product and a primary source of income for farmers. However, consumer acceptance of meat products is largely influenced by their quality. Notably, melatonin has also shown positive effects on the quality of meat across various animal species. In broiler chicken incubation, monochromatic green light stimulation increases melatonin secretion, promoting GH activity and the IGF-1 axis. This activation is crucial for stimulating muscle satellite cell proliferation, muscle fiber formation, and overall muscle development, involving the gene expression of paired box 7 (PAX7) and myogenic regulatory factors. These factors contribute to the improvement of broiler growth and meat quality (214).

In pigs, melatonin supplementation enhances the cross-sectional area of weaned piglet muscle fibers and the weight of the longest back and eye muscle areas. Additionally, it reduces the triglyceride levels in the longest back and loin muscles. Melatonin further promotes muscle fiber development (upregulating *PAX7*, *MTOG*, and *IGF-1*) and reduces fat deposition in muscles (downregulating *PPARγ*, *ACC*, and *FABP4*) by modulating gene expression. Moreover, melatonin suppresses intramuscular fat cell proliferation by activating the MT2-mediated PKA/ERK1/2 signaling pathway (104, 105).

In ruminants, melatonin has limited effects on the carcass quality of cashmere goats and does not adversely impact meat production, composition, or quality (215). Studies have detected melatonin in meat, with chicken eggs showing the highest levels. Melatonin extends the shelf life of meat, preserving its quality and taste (216). Therefore, the consumption of these foods by humans and animals may offer potential health benefits. Although research on the role of melatonin in meat quality is not extensive, it shows promise in enhancing poultry and pork quality and extending shelf life.

## 5 General discussion

Following the prohibition on the use of antibiotics as feed additives, the search for alternative methods to sustain animal health and enhance production efficiency has become imperative. Although research on the impact of melatonin on animal production performance is limited, studies have revealed its moderately favorable effects on growth rate, weight gain, and feed conversion efficiency. The primary advantages of melatonin lie in its ability to enhance reproductive performance, exert antioxidative and immunoregulatory effects, improve disease resistance, and contribute to the overall health of animals.

In poultry, leveraging melatonin through green light exposure has shown promise in stimulating melatonin secretion and benefiting poultry health. In animal production, melatonin is typically considered a component of supplementary healthcare and health management rather than a primary means to boost production performance. However, a comprehensive understanding of the diverse biological activities of melatonin and its structural–functional relationship remains incomplete. Further exploration is required to determine the extent of the applicability of melatonin and the optimal conditions for its use, considering potential variations in responses across different animal species and growth stages.

Moreover, investigating the interactions between melatonin and other feed components is crucial to comprehending its synergistic effects with various ingredients and optimize feed formulations more efficiently. The primary limitation of this review lies in the existing heterogeneity across studies, encompassing variations in methodology, animal species, and environmental conditions. The scarcity of research specifically dedicated to melatonin’s impact on animal production further adds complexity, limiting the ability to draw universally applicable conclusions. Additionally, the concentration of current research in the medical domain, particularly in humans and mice, poses challenges in directly extrapolating findings to the diverse contexts of animal farming. However, with mounting interest in the potential use of melatonin in animal production, future research holds promise for providing more robust scientific evidence to support its widespread application and alignment with the needs of the animal farming industry.

## 6 Conclusions and perspectives

Overall, melatonin exhibits specific biological characteristics and physiological functions in animal farming, with the potential to serve as an alternative to antibiotics. The outcomes of our review underscore the need for targeted research efforts to unlock the full potential of melatonin in enhancing animal well-being and farm productivity. Looking ahead, researchers should focus on elucidating the mechanisms underlying melatonin’s effects, exploring optimal administration methods, and assessing its long-term impacts on different aspects of animal health and performance. By addressing these research gaps, we can harness the capabilities of melatonin to their fullest extent, paving the way for sustainable and effective practices in animal production.
